# Toxicological Assessment of *Origanum majorana* L.: Evaluation of Its Cytotoxicity, Genotoxicity, and Acute Oral Toxicity

**DOI:** 10.3390/ijms26199461

**Published:** 2025-09-27

**Authors:** Ayfer Beceren, Ayse Nur Hazar-Yavuz, Ozlem Bingol Ozakpinar, Duygu Taskin, İsmail Senkardes, Turgut Taskin, Ozlem Tugce Cilingir-Kaya, Ahmad Kado, Hatice Kubra Elcioglu

**Affiliations:** 1 Department of Pharmaceutical Toxicology, Faculty of Pharmacy, Marmara University, Istanbul 34854, Türkiye; 2 Department of Pharmacology, Faculty of Pharmacy, Marmara University, Istanbul 34854, Türkiye; ayse.hazar@marmara.edu.tr (A.N.H.-Y.); kubra.elcioglu@marmara.edu.tr (H.K.E.); 3 Department of Biochemistry, Faculty of Pharmacy, Marmara University, Istanbul 34854, Türkiye; ozlem.bingol@marmara.edu.tr; 4 Marmara Pharmacy Drug and Innovative Product Development Unit, Faculty of Pharmacy, Marmara University, Istanbul 34854, Türkiye; duygu.taskin@sbu.edu.tr (D.T.); turguttaskin@marmara.edu.tr (T.T.); 5 Department of Analytical Chemistry, Faculty of Pharmacy, University of Health Sciences, Istanbul 34668, Türkiye; 6 Department of Pharmaceutical Botany, Faculty of Pharmacy, Marmara University, Istanbul 34854, Türkiye; isenkardes@marmara.edu.tr; 7 Department of Pharmacognosy, Faculty of Pharmacy, Marmara University, Istanbul 34854, Türkiye; 8 Department of Histology and Embryology, School of Medicine, Marmara University, Istanbul 34854, Türkiye; tugce.cilingir@marmara.edu.tr; 9 ISLAB-2 Core Laboratory, Goztepe Prof. Dr. Suleyman Yalcin City Hospital, Istanbul 34722, Türkiye; ahmetkado94@hotmail.com

**Keywords:** *Origanum majorana* L., acute toxicity, haematological analysis, biochemical analysis, histological analysis

## Abstract

Medicinal plants remain central to traditional healthcare, yet their increasing integration into modern pharmacology necessitates robust toxicological evaluation. *Origanum majorana* L. (sweet marjoram), widely used in culinary and folk medicine, contains diverse secondary metabolites with both therapeutic and potential genotoxic activities. Despite its popularity, systematic in vivo and in vitro safety assessments remain limited. This study aimed to comprehensively evaluate the acute oral toxicity, cytotoxicity, and genotoxicity of *O. majorana* methanolic extract, providing baseline toxicological data to support its safe traditional use and potential pharmaceutical applications. The methanol extract of *O. majorana* leaves was tested in NIH-3T3 fibroblasts for cytotoxicity and genotoxicity. In vivo acute oral toxicity was assessed in rats according to OECD Guideline 420, with animals monitored over 14 days for clinical signs, hematological and biochemical alterations, and histopathological changes. The extract preserved fibroblast viability above 90% across all tested concentrations (10–200 µg/mL), indicating absence of cytotoxicity. However, comet and micronucleus assays revealed dose-dependent DNA damage, suggesting genotoxic potential at higher exposures. In vivo, no mortality or overt systemic toxicity was observed at doses up to 2000 mg/kg. Hematological analyses showed immunomodulatory shifts (increased neutrophils and monocytes, reduced eosinophils), while biochemical profiles indicated hepatoprotective and cardioprotective effects, with reduced ALT, AST, and LDH levels. Histopathological evaluation revealed only mild, focal changes consistent with adaptive rather than irreversible responses. *O. majorana* extract demonstrates a favorable acute safety profile with preserved hepatic and renal function, hematological modulation, and absence of in vitro cytotoxicity. Nevertheless, dose-dependent genotoxicity warrants caution for concentrated formulations. According to GHS classification, the extract aligns with Category 5 (acute oral toxicity, lowest hazard) and Category 2 (germ cell mutagenicity). These findings underscore the importance of dose management and further long-term genotoxicity studies before translational applications in nutraceutical or biomedical fields.

## 1. Introduction

Medicinal plants have long shaped traditional healing systems. In many parts of the world, especially where modern medical services are scarce, they remain the backbone of primary healthcare. However, the increasing interest in phytotherapeutics and integration of such plants into contemporary pharmacological formulations demand a clear understanding of their safety profiles [1]. This becomes notably important when considering the complex composition of plant matrices, which may contain bioactive compounds with both therapeutic and toxic potential.

Toxicological studies, especially those investigating acute toxicity, cytotoxicity, and genotoxicity, are fundamental for evaluating the safety of traditionally used medicinal plants. Such studies are indispensable when these botanicals are considered for broader pharmacological applications or incorporation into herbal products [2].

According to taxonomic revisions [3,4], the genus *Origanum* L. comprises ten sections, nine of which are predominantly distributed across Greece, the southern Balkans, and the eastern Mediterranean. Historically, *Origanum* species have been central to both culinary and medicinal traditions. Moreover, their economic and cultural value has persisted for centuries. In modern times, these aromatic plants continue to be extensively utilized in the agricultural, pharmaceutical, and cosmetic sectors, where they are valued for their secondary metabolites with pharmacological activity and significant commercial potential [5,6].

*Origanum majorana* L. (*O. majorana*) is found in the flora of Türkiye, especially in Southern Anatolia [7]. The plant, commonly known as ‘sweet marjoram’, is referred to as ‘Mercanköşk’ in Türkiye, as is the case with other *Origanum* species; additionally, it is known by various local names such as ‘Kekik otu’, ‘Göğe kekiği’, ‘Kahve otu’, ‘Guy otu’, ‘Sebso’, and ‘Sebzo’ [8,9]. *O. majorana* is traditionally used as a culinary spice and in folk medicine. The flowering and leafy branches of *O. majorana* have been reported to be used in Anatolia as a folk medicine, particularly in the form of decoctions and infusions, for the relief of colds, coughs, flu, sore throat, stomach ache, indigestion, gastric ulcers, nausea, intestinal gas, and diarrhea. They are also traditionally employed as a diuretic and for the treatment of urinary tract diseases, as well as for their sedative effects and in the management of diabetes, hypertension, arteriosclerosis, gallbladder diseases, and to promote sweating [10]. Its essential oil is characterized by high levels of terpinen-4-ol, often accompanied by cis-sabinene hydrate, γ-terpinene, α-terpineol, linalool, and p-cymene [11]. Depending on chemotype and geographic origin, minor amounts of thymol and carvacrol may also occur [12]. These volatile compounds have been associated with antimicrobial, antioxidant, and anti-inflammatory properties, making *O. majorana* a valuable source of bioactive metabolites and a subject of growing pharmacological interest [13].

Despite its growing culinary and medicinal use, detailed in vivo toxicity data on *O. majorana* remain scarce, an omission that may lead to overestimating its safety in routine consumption. While low doses of carvacrol can be beneficial, higher concentrations have been linked to adverse outcomes, including DNA damage in certain experimental settings [14]. Thus, understanding the dose–response relationship and potential cytotoxic or genotoxic risks associated with *O. majorana* is crucial, particularly for populations using it frequently or in combination with conventional drugs.

The present study examines the acute oral toxicity of *O. majorana* methanol extract in mice, following OECD Test Guideline No. 420 [15]. It also evaluates in vitro cytotoxicity and genotoxicity via MTT, comet, and micronucleus assays in NIH3T3 cells (a standard non-tumorigenic murine cell line widely used for toxicity screening), aiming to provide a comprehensive safety assessment. These evaluations are intended to establish baseline toxicological data for *O. majorana*, supporting its traditional use while ensuring a scientific basis for potential pharmaceutical applications.

## 2. Results

### 2.1. Phenolic Compounds Analysis Results

**Figure 1:**
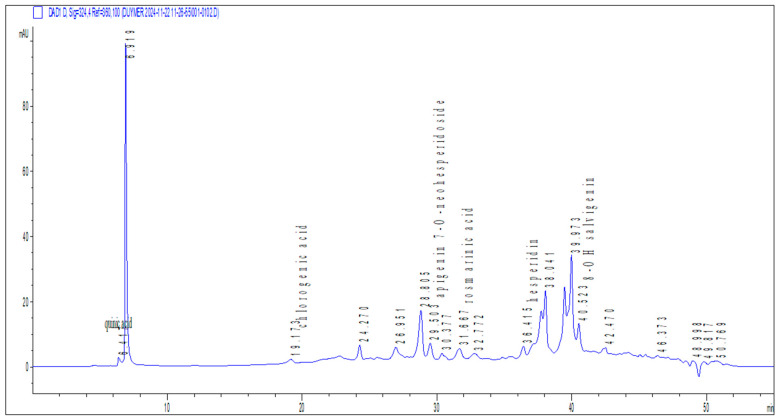
HPLC-DAD chromatogram of extract at 330 nm.

The phytochemical content of the methanol extract derived from the *O. majorana* was determined qualitatively by HPLC-DAD system (Figure 1). In this study, six phenolic compounds were identified in the methanol extract including quinic acid, chlorogenic acid (CGA), apigenin-7-O-neohesperidoside, rosmarinic acid, hesperidin and 8-OH salvigenin (Table 1).

### 2.2. Cytotoxicity

**Figure 2:**
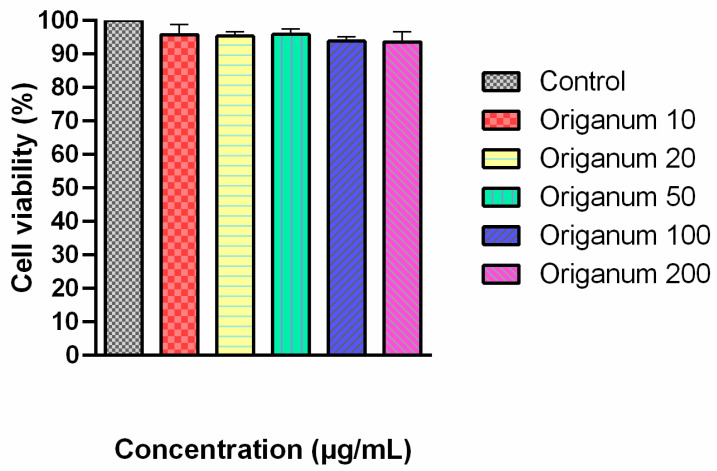
Dose-dependent evaluation of *O. majorana* extracts on cell viability.

Results from the MTT assay indicate that treatment with *O. majorana* extract at concentrations ranging from 10 to 200 µg/mL did not significantly affect cell viability compared to the control group (Figure 2). Cell viability remained above 90% at all concentrations tested, indicating that the extract did not exhibit cytotoxic effects in this range. These findings demonstrate that *O. majorana* extract was well tolerated by the cells under in vitro conditions and can be considered safe for further biological or pharmacological evaluations.

### 2.3. Genotoxicity

#### 2.3.1. Comet Assay Results

**Figure 3:**
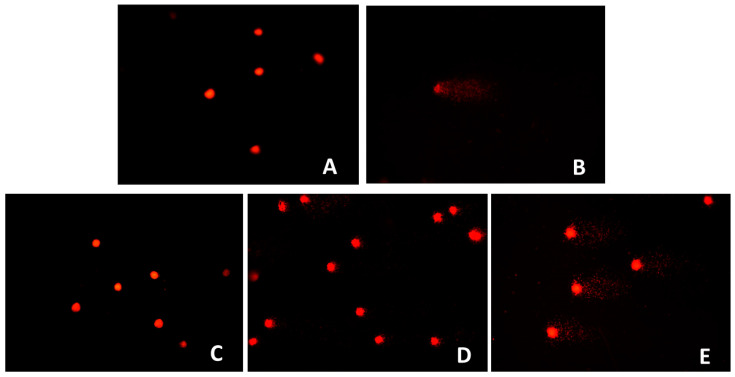
Images of comet assays in fibroblasts subjected to methanolic extracts from *O. majorana* at 400× magnification. (**A**) Control, (**B**) Positive control (50 µM H_2_O_2_), (**C**) *O. Majorana extract* (10 µg/mL), (**D**) *O. majorana* extract (100 µg/mL), (**E**) *O. majorana* extract (200 µg/mL).

The comet assay demonstrated a statistically highly significant increase in DNA damage (%DNA_T_) at *O. majorana* extract concentrations of 100 and 200 µg/mL compared to the control group (*p* < 0.001), whereas the 10 µg/mL concentration did not differ significantly from the control (*p* > 0.05). Notably, all extract-treated groups exhibited significantly lower DNA damage levels than the positive control (*p* < 0.001) and indicating that *O. majorana* exerts a protective effect against oxidative DNA damage, with only mild genotoxic potential observed at higher concentrations (Table 2; Figure 3).

#### 2.3.2. MN Test Results

In the MN assay, treatment with *O. majorana* extract resulted in a dose-dependent elevation at the MN frequency (Table 3). At the lowest concentration (10 µg/mL) tested, no significant difference was observed compared with the control group. However, exposure to 100 µg/mL (*p* < 0.01 vs. control) and 200 µg/mL (*p* < 0.001 vs. control) produced a statistically significant increase in MN formation. Despite this rise, all concentrations of the extract induced markedly lower MN frequencies than the positive control, mitomycin C (*p* < 0.001), which itself caused a pronounced elevation relative to control (*p* < 0.001). In parallel, analysis of the CBPI revealed no significant alteration at 10 µg/mL, whereas a significant reduction was detected at 100 and 200 µg/mL (*p* < 0.001 vs. control), indicating a concentration-dependent inhibition of cell proliferation at higher doses.

Both the comet and MN assays revealed dose-dependent genotoxic effects of *O. majorana* extract. The comet assay showed a significant increase in DNA strand breaks at concentrations of 100 and 200 µg/mL concentrations (*p* < 0.001), while the MN assay showed a significant increase in MN frequency at the same concentrations (*p* < 0.01 and *p* < 0.001, respectively).

At the lowest concentration tested (10 µg/mL), neither assay showed any statistically significant changes compared to the control group, except for a minimal and nonsignificant increase.

It is noteworthy that the comet assay provides a more sensitive earlier indicator of genotoxic events by detecting primary DNA strand breaks, while the MN assay represents a more advanced stage of genomic instability by reflecting chromosomal damage or loss. The concordant findings from both analyses, taken together, highlight the genotoxic potential of *O. majorana* extracts. Specifically, the results show that DNA damage initially detected by the comet assay can manifest as chromosomal abnormalities that can later be detected by the MN test.

### 2.4. Results of Acute Toxicity Study

#### 2.4.1. Sighting Study

The sighting study revealed no observable signs of toxicity, including backward walking, stomach walk, tremor, diarrhea, salivation, and weakness, at any of the administered dose levels (5, 50, 300, and 2000 mg/kg BW). All experimental animals survived throughout the observation period. Based on these findings, we then selected dose levels of 300 and 2000 mg/kg BW for the main study.

#### 2.4.2. Main Study

Administrations of *O. majorana* extract at fixed doses of 300 and 2000 mg/kg BW did not result in treatment-related mortality at the 300 mg/kg dose. At the higher dose of 2000 mg/kg, however, one animal died during the 14-day observation period. Apart from this single case at 2000 mg/kg, no additional deaths occurred, and the surviving rats did not display clinical signs of acute toxicity. Food and water intake remained unaffected, and no manifestations such as salivation, aggression, piloerection, or writhing were detected.

#### Physical Observation and Mortality

The acute toxicity study indicated that administration of *O. majorana* extract at a dose of 2000 mg/kg resulted in mortality in one animal, whereas no deaths occurred at the 300 mg/kg dose. During the 14-day observation period, the surviving treated rats did not exhibit overt signs of toxicity, such as alterations in skin, fur, eyes, and mucous membranes, as well as behavioural abnormalities, tremors, salivation, diarrhea, disturbances in sleep patterns, or coma.

#### Acute-Effect on BW, Food and Water Consumption

Table 4 presents the alterations in the mean values of BW, food consumption, and water intake of female rats administered 300 and 2000 mg/kg of methanol extract of *O. majorana* on the first day and subsequently on days 7 and 14 in compared with the control group. The administration of *O. majorana* extracts at dosages of 300 and 2000 mg/kg BW to rats led to a gradual increase in BW over time, comparable to the control group, suggesting that the extracts exhibited no acute toxicity. Food and water intake in the treatment group did not differ significantly from that of the control group throughout the duration of study.

#### Haematological Parameters of Acute Toxicity

Hematological analysis demonstrated distinct and dose-dependent alterations following administration of *O. majorana* extract. At 300 mg/kg dose, neutrophil counts were markedly elevated (*p* < 0.01), accompanied by significant increases in monocytes (*p* < 0.001) and immature granulocytes (*p* < 0.05). In contrast, eosinophil counts and percentages decreased substantially (*p* < 0.01 and *p* < 0.001, respectively), whereas the neutrophil percentage was significantly higher compared to controls (*p* < 0.05). At 2000 mg/kg dose, a comparable pattern emerged, with reductions in eosinophil and basophil counts (*p* < 0.01–0.001) and a decrease in immature granulocyte percentages (*p* < 0.05–0.01).

Red blood cell indices remained largely stable, except for significant elevations in MCHC (*p* < 0.001 at 300 mg/kg; *p* < 0.05 at 2000 mg/kg) and RDW parameters (*p* < 0.05 and *p* < 0.001), suggesting subtle effects on erythrocyte morphology. The most pronounced and consistent alterations were observed in platelet indices: platelet counts increased markedly in both treated groups (*p* < 0.001), whereas MPV, PDW, and P-LCR values were significantly reduced (*p* < 0.001).

Overall, these results indicate that *O. majorana* extract exerts measurable hematological effects, particularly within leukocyte subsets and platelet indices, while preserving the stability of the most erythrocyte parameters. Such findings may reflect immunomodulatory and thrombopoietic influences of the extract, warranting further investigation into underlying mechanisms (Table 5).

#### Effects of Plant Extracts on Biochemical Markers

Oral administration of *O. majorana* extract at both 300 mg/kg (*O. majorana* I) and 2000 mg/kg (*O. majorana* II) produced dose-dependent alterations across several biochemical parameters, reflecting organ-specific modulation without clear evidence of systemic toxicity.

In the hepatic profile, ALT and AST activities were significantly reduced in both treatment groups (*p* < 0.001), which may indicate hepatoprotective or regulatory effects on enzymatic activity. Conversely, ALP levels increased markedly (*p* < 0.01–0.001), suggesting a selective influence on biliary or bone-related pathways. Total protein and albumin concentrations remained unchanged, indicating preserved liver synthetic capacity despite enzymatic variations.

Renal function markers including urea, BUN, and creatinine remained stable, supporting maintained glomerular and tubular function. In contrast, UA and Mg levels decreased significantly in a dose-dependent manner (*p* < 0.05–0.001), and phosphate levels were markedly reduced at the higher dose (*p* < 0.001). These changes suggest an influence on mineral metabolism, although electrolyte balance was largely preserved, with only a modest increase in chloride at the lower dose (*p* < 0.05).

In the cardiac profile, LDH activity was significantly reduced at the higher dose (*p* < 0.01), while amylase levels exhibited a dose-dependent decline (*p* < 0.05). CK levels varied but did not reach statistical significance, and additional metabolic markers (CO_2_, NH_3_, lipase) showed only minimal changes, together suggesting an absence of overt cardiac or metabolic toxicity.

Within the lipid profile, a significant reduction in triglyceride levels was observed in both treatment groups (*p* < 0.05–0.01), whereas total cholesterol and glucose remained unchanged, indicating a potential lipid-lowering effect of the extract without disturbing glycemic regulation.

Overall, these findings demonstrate that *O. majorana* extracts exert measurable and dose-dependent effects on hepatic enzymes, renal mineral balance, and lipid metabolism, while maintaining general biochemical stability. Subtle alterations in ALP, magnesium, phosphate, and uric acid emphasize the need for dose-dependent monitoring and further mechanistic studies to clarify the long-term implications of these changes (Table 6).

#### Histological Analyses

The relative organ weight of control and rats treated with *O. majorana* extract measured by the acute toxicity study (Table 7). The relative organ weights of rats treated with *O. majorana* extract at both 300 mg/kg (*O. majorana* I) and 2000 mg/kg (*O. majorana* II) doses did not exhibit statistically significant differences compared to the control group (*p* > 0.05). Lung, liver, heart, spleen, kidneys, and ovaries maintained consistent relative weights across all experimental groups, indicating that acute administration of the extract did not induce organ hypertrophy or atrophy. These findings suggest that *O. majorana* extract does not exert overt organotoxic effects under the conditions tested in this acute toxicity study.

**Figure 4:**
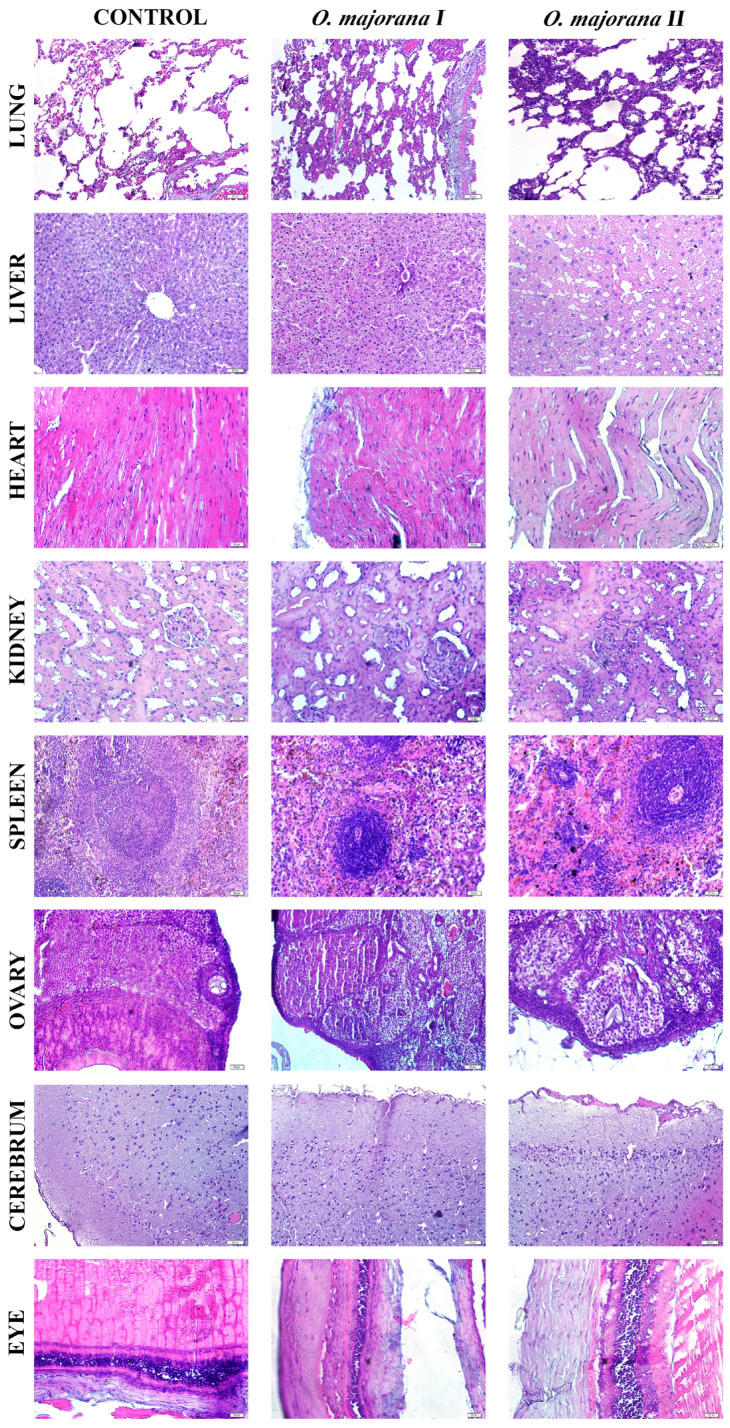
Representative micrographs of the lung, liver, heart, kidney, spleen, ovary, cerebrum, and eye tissues from the control (left column), *O. majorana* I (300 mg/kg; middle column), and *O. majorana* II (2000 mg/kg; right column) experimental groups. Haematoxylin and eosin (H&E); 20× magnification. The scale bar is 50 µm in all images.

Histological assessment of tissue samples collected from rats treated with *O. majorana* extract at doses of 300 mg/kg and 2000 mg/kg BW revealed mild to moderate changes in some organs, while others retained near-normal architecture (Figure 4). The semi-quantitative scores are summarized in Table 8.

- Lung: Normal alveolar structure in the control group; mild septal thickening and limited leukocyte infiltration were noted at higher doses.
- Liver: Hepatocyte cords and sinusoidal architecture appeared normal in the control group. Sinusoidal congestion, hepatocellular vacuolization, and focal inflammatory cell infiltration were more prominent in the groups treated with increasing extract doses.
- Heart: The control cardiac muscle showed compact and regular striations, whereas myofiber disorganization and sarcoplasmic rarefaction were observed.
- Kidney: The control kidneys showed mild tubular dilation and preserved glomeruli. Tubular dilation and cytoplasmic eosinophilia increased in a dose-dependent manner in the kidney.
- Spleen: Control spleen samples showed intact white and red pulp zones in the spleen. White pulp hyperplasia and follicular disorganization were observed in the treatment groups.
- Ovary: Follicular development and stromal architecture were preserved in the controls. Stromal oedema and degenerating follicles were visible, exhibiting dose-dependent increases.
- Cerebrum: Cortical lamination was intact in the control animals. Disruption of cortical lamination, neuronal shrinkage, and mild gliosis were observed in the post-treatment groups.
- Eyes: Mild epithelial irregularities were observed in the control group. Stromal disarray, epithelial thinning, and stromal disorganization were more evident in treated groups, although no retinal detachment or haemorrhage was observed.

## 3. Discussion

The present study provides a multifaceted evaluation of *O. majorana* methanolic extract, integrating phytochemical profiling with in vitro cytotoxicity and genotoxicity assays, alongside acute in vivo toxicity assessments in a rodent model. The findings collectively indicate a complex pharmacological profile, where beneficial antioxidant constituents coexist with dose-dependent genotoxic signals at higher concentrations, though without overt systemic toxicity in vivo.

*O. majorana* is rich in bioactive polyphenolic compounds and flavonoids including rosmarinic acid, apigenin derivatives, luteolin, and chlorogenic acid which have well-documented antioxidant, anti-inflammatory, and organ-protective effects [16,17,18]. Rosmarinic acid, in particular, promotes hepatoprotection through Nrf2 activation and suppression of oxidative stress pathways [19]. Additionally, hesperidin and related flavonoids display beneficial lipid-modulating and anti-inflammatory properties, offering a mechanistic basis for the metabolic and hematological improvements as observed in this study [20].

The phytochemical analysis revealed apigenin-7-O-neohesperidoside as the predominant compound in the *O. majorana* extract. Previous studies have highlighted its anticancer and cytotoxic properties, suggesting potential therapeutic roles in targeting proliferative disorders [21]. Interestingly, despite its reported cytotoxic effects against cancerous cells, our in vitro findings showed no significant cytotoxicity toward non-cancerous NIH3T3 fibroblasts, even at the highest concentration tested. This apparent selectivity for malignant cells over normal ones aligns with literature proposing apigenin-7-O-neohesperidoside as a low-toxicity candidate for anticancer drug development, warranting further investigation into its differential cellular mechanisms.

Similarly, rosmarinic acid, another major phenolic constituent identified in our extract, has been associated with hepatoprotective activity in experimental models [22]. Consistent with this, we observed significant reductions in hepatic enzymes (ALT, AST) and the absence of overt liver toxicity in treated animals, even at the highest administered dose. These findings collectively suggest that *O. majorana*’s phytochemical profile may confer protective effects on hepatic tissue, potentially mitigating oxidative or inflammatory insults while exhibiting genotoxic effects only at supratherapeutic concentrations.

In addition, CGA, also detected in methanol extract, may contribute to the metabolic and hepatic improvements observed in this study. Previous studies have shown that CGA significantly reduces ALT and AST levels and suppresses oxidative stress and inflammatory pathways in various hepatic injury models [23], supporting our findings of lowered hepatic enzyme activities and preserved tissue morphology. Moreover, CGA has been reported to enhance lipid metabolism via inhibition of the FXR/FGF15 signaling pathway and upregulation of CYP7A1 expression, thereby promoting cholesterol catabolism and triglyceride clearance [24]. These mechanistic insights align with the reduced serum triglycerides and stable glycemic profiles observed in the present study, further highlighting CGA as a key contributor to the extract’s beneficial metabolic effects. Consistently, Lieshchova and Brygadyrenko reported that dietary supplementation with *Origanum vulgare* significantly reduced serum triglycerides, lowered the atherogenic index, increased HDL-cholesterol, and improved glycemic control in high-fat diet–induced obese rats [25]. These findings complement our results by demonstrating that *Origanum* species can beneficially modulate lipid and glucose metabolism in vivo. Taken together, the hepatoprotective and lipid-lowering properties observed in our study with *O. majorana* are in strong agreement with the independent evidence provided for *O. vulgare*, further supporting the therapeutic potential of *O. majorana* extract in the management of metabolic disorders.

Importantly, *O. majorana* specific studies corroborate our chemical and biological readouts. Targeted HPLC-DAD/ESI-MS profiling consistently identifies rosmarinic acid as predominant, with 5-caffeoylquinic (chlorogenic) acid and apigenin glycosides also present in ethanolic extract [26]. Functionally, *O. majorana* aqueous and organic fractions produce dose-dependent cytotoxicity in cancer cell lines (MDA-MB-231, HT-29) while showing no acute oral toxicity at 5–10 g/kg over 14 days in mice paralleling our observation that no acute oral toxicity over 14 days in rats and normal NIH3T3 fibroblasts retain viability across 10–200 µg/mL [27]. Consistently, a hydroethanolic extract evaluated under OECD 423 showed no acute toxicity at 2 g/kg with normal clinical observations during 14-day follow-up, supporting a wide acute safety margin in vivo [28].

With respect to genetic safety, the matrix and dose clearly matter. While our phenolic-rich methanolic extract yielded comet/MN signals at upper in vitro concentrations, *O. majorana* essential oil (terpenoid-rich, compositionally distinct) was Ames-negative and V79 MN-negative at non-cytotoxic doses [29]. Together with our acute rat data, these reports suggest that genotoxic alerts are assay- and concentration-dependent, and they motivate subacute/subchronic in vivo genotoxicity (e.g., bone-marrow MN, organ-specific comet) to determine translational risk at realistic exposure levels.

The cytotoxicity assay demonstrated that *O. majorana* extracts maintained NIH3T3 fibroblast cell viability above 90% across all tested concentrations (10–200 µg/mL) after 24 h of exposure, confirming the absence of direct cytotoxic effects within this dose range. These findings are consistent with previous evidence that rosmarinic acid, a predominant phenolic compound in *O. majorana*, protects mammalian cells against oxidative stress and apoptosis rather than inducing cytotoxicity at moderate concentrations [19]. Similarly, hesperidin and related flavonoids have been shown to exert cytoprotective and anti-apoptotic effects in vitro, further supporting the biocompatibility observed in our study [20,30]. Although there is limited information on in vivo toxicity, several in vitro studies have reported that *O. majorana* extracts and their essential oils exhibit dose-dependent cytotoxicity against various cancer cell lines, including breast, colon, and lung carcinoma, generally through induction of apoptosis and oxidative stress [31,32,33]. More importantly, these effects were more pronounced in malignant cells than in normal cells, indicating some degree of selectivity and supporting the safety profile observed in our fibroblast model [31,34]. Collectively, these results highlight the safety of *O. majorana* extracts under basal conditions and strengthen their potential for safe application in biomedical and nutraceutical formulations.

In contrast, both the comet and MN assays revealed a clear dose-dependent genotoxic effect of *O. majorana* extract for 24 h at higher concentrations (100 and 200 µg/mL), with increased DNA strand breaks and elevated MN formation observed. These results suggest that while the extract may not be cytotoxic at these concentrations, it has the potential to compromise genomic integrity under conditions of high exposure. Evidence from a broad review of medicinal plants supports this observation, indicating that DNA damage is a common risk associated with certain herbal preparations especially within the Lamiaceae family when tested in genotoxicity assays [35]. This consistency across test methodologies underlines the necessity of establishing strict dose limits and safety thresholds for the therapeutic use of *O. majorana* extracts in nutraceuticals and clinical applications.

In vivo acute toxicity outcomes demonstrated compelling protective effects of *O. majorana* on key hematological and biochemical parameters. Notably, at doses of 300 mg/kg and 2000 mg/kg, we observed elevations in neutrophil and monocyte counts, reductions in eosinophils, and modulated platelet indices. These hematological shifts align with prior evidence, as Ramadan et al. reported that oral administration of *O. majorana* leaf extracts significantly attenuated granulocytosis and thrombocytosis in isoproterenol-induced cardiac injury models indicating genuine immunomodulatory potential rather than non-specific toxicity [36]. Biochemically, our findings revealed substantial reductions in serum ALT, AST, and LDH levels, suggesting hepatoprotective and cardioprotective roles for the extract. Ramadan et al. similarly documented decreases in troponin, LDH, and aminotransferases in treated rats, which they attributed to the antioxidant effects of *O. majorana* phytochemicals [36]. The overlapping patterns between these works reinforce that the observed hematologic normalization and enzyme modulation are likely linked to *O. majorana*’s rich antioxidant profile.

Histopathological examination revealed that acute administration of *O. majorana* extract caused mild to moderate morphological alterations in certain organs, despite the absence of gross toxicity as evidenced by unchanged relative organ weights (*p* > 0.05). Specifically, hepatic tissues exhibited sinusoidal congestion, hepatocellular vacuolization, and focal inflammatory infiltrates, while renal sections showed tubular dilation and cytoplasmic eosinophilia with preserved glomerular architecture. These findings indicate that although biochemical markers such as ALT and AST suggested hepatoprotection, microscopic changes point to subtle hepatic stress, consistent with reports in related *Origanum* species [37].

Renal histological changes corresponded with normal renal biochemical markers (urea, creatinine, BUN), suggesting adaptive responses rather than overt nephrotoxicity. Moreover, previous studies demonstrated that *Origanum* species exert significant nephroprotective and antioxidant effects against chemical-induced renal injury, further corroborating our findings [38]. Splenic white pulp hyperplasia and follicular disorganization aligned with hematological findings of increased neutrophils and monocytes and reduced eosinophils, supporting an immunomodulatory role of flavonoids such as apigenin and hesperidin. Cardiac muscle showed mild myofiber disorganization and sarcoplasmic rarefaction, which, when considered alongside decreased LDH levels, may reflect cardioprotective effects of rosmarinic acid and related polyphenols. Other tissues, including ovary, cerebrum, and eye, exhibited dose-dependent mild changes such as stromal edema, neuronal shrinkage, and epithelial thinning, but without significant biochemical disturbances, indicating transient adaptive responses rather than irreversible damage. Overall, histopathological observations complement hematological and biochemical results, suggesting that *O. majorana* extract exerts functional protection at the systemic level while inducing limited microscopic stress responses in certain organs.

Consistently with stable organ weights and improved biochemistry (ALT/AST, LDH), the mild, focal histological changes are most compatible with subclinical/adaptive responses after acute exposure. To consolidate structure–function alignment, we will provide blinded semi-quantitative scoring with inter-rater documentation and correlate individual scores with biochemical measures. A brief subacute follow-up (both sexes), including pathway markers (hepatic Nrf2/HO-1, FXR–FGF15/CYP7A1; renal KIM-1/NGAL), will clarify persistence or reversibility and further support the protective profile.

Taken together, these data depict a dualistic profile: while *O. majorana* methanol extract are safe and protective in terms of organ function, oxidative balance, and hematological modulation at low to moderate exposures, they exhibit genotoxic potential in vitro at higher concentrations. This dichotomy underscores the importance of dose in determining the balance between therapeutic and adverse effects. Importantly, the traditional dietary use of *O. majorana* suggests that low-level exposures are safe, but concentrated extracts for clinical or nutraceutical use require rigorous safety evaluation. Future work should integrate long-term in vivo genotoxicity assays and mechanistic studies on DNA repair pathways to define evidence-based safety thresholds.

## 4. Materials and Methods

### 4.1. Data Collection and Identification of Plant Sample

The plant samples of the *O. majorana* were collected from Yalova/Türkiye and taxonomically described using Flora of Turkey and the East Aegean Islands [7] by Dr. Ismail Senkardes. Also, the scientific names of plant taxa were checked and updated according to World Flora Online [39]. A voucher specimen of the aerial parts of *O. majorana* was stored at the Herbarium of the Faculty of Pharmacy at Marmara University (voucher number: MARE-23195).

### 4.2. Preparation of Plant Extract

The identified plant was dried in a cool, sun-protected room, and then a methanol extract was prepared using maceration. The extraction was continued until the solvent became colorless and resulting methanol solvents were subsequently filtered through filter paper and concentrated using a rotary evaporator (Heidolph Hei VAP, Heidolph Instruments GmbH & Co. KG, Schwabach, Germany) at low pressure and 45 °C. The methanol extracts were stored in a refrigerator at 4 °C [40].

### 4.3. Analysis of Phenolic Compounds

Phenolic compounds in the methanol extract obtained from the plant extract were determined using the high-pressure liquid chromatography (HPLC-DAD) system 1260 Infinity II (Agilent Technology, Santa Clara, CA, USA) using the method previously applied by our team [41].

### 4.4. Cytotoxicity Assay

The cytotoxic potential of *O. majorana* extract was assessed using the mouse embryonic fibroblast cell line, NIH3T3 (ATCC CRL-1658). Cells were cultured in Dulbecco’s Modified Eagle Medium/Nutrient Mixture F-12 (DMEM/F12, Gibco, Life Technologies Limited, Paisley, UK) supplemented with 10% fetal bovine serum, 1% L-glutamine, and penicillin–streptomycin and maintained at 37 °C in a humidified atmosphere containing 5% CO_2_.

For the assay, cells were seeded at a density of 1 × 10^4^ cells per well in 96-well plates and incubated overnight for cell attachment. At the end of the incubation period, cells were exposed to different concentrations of the extract (10–200 µg/mL) for 24 h. Cell viability was determined using the 3-(4,5-dimethylthiazol-2-yl)-2,5-diphenyltetrazolium bromide (MTT) assay, as described previously [42]. After incubation, MTT solution (5 mg/mL) was added to each well and incubated for an additional 4 h. Then, the medium was carefully aspirated and the formazan crystals were dissolved in SDS buffer.

Absorbance values were measured using a microplate spectrophotometer (BioTek Instruments, Winooski, VT, USA) at 570 nm at a reference wavelength of 630 nm. All treatments were conducted in triplicate and the experiments were independently repeated twice to ensure reproducibility. Cell viability was calculated according to the following equation:

% Cell Viability = [(Mean OD of treated cells)/(Mean OD of control cells)] × 100

### 4.5. Evaluation of In Vitro Genotoxicity

#### 4.5.1. Comet Assay

The alkaline comet assay was applied to determine the genotoxic potential of *O. majorana* extracts on NIH3T3 cells. Cells (1 × 105/well) were seeded in 6-well plates and treated with final concentrations of 10, 100, and 200 µg/mL for 24 h. After incubation, cells were trypsinized, resuspended in medium, and subjected to the comet assay following a slightly modified protocol of Singh et al. [43]. Cells were embedded in low-melting agarose on slides precoated with high-melting agarose, solidified at 4 °C for 15 min, and lysed at 4 °C for 1 h.

Following lysis, slides were rinsed with precooled distilled water, placed in alkaline buffer (300 mM NaOH, 1 mM EDTA) for 20 min to unwind DNA, and electrophoresed at 300 mA and 15 V for 20 min. Slides were neutralized with Tris buffer (0.4 M, pH 7.5), stained with ethidium bromide, and examined under a fluorescence microscope at 400× magnification (Olympus BX51, Tokyo, Japan). DNA damage, expressed as the percentage of DNA in the tail (%DNAT), was determined by scoring 100 cells (50 per slide) using BAB Bs200Pro software (BAB Ltd., Ankara, Turkey), with untreated cells as the negative control and 50 µM (1.7 µg/mL) H_2_O_2_ as the positive control [44]. All experiments were performed in triplicate, and results are presented as mean ± SD.

#### 4.5.2. Micronucleus (MN) Test

The micronucleus (MN) assay was performed on NIH3T3 cells following Fenech [45]. Cells (2 × 10^5^/well) were seeded in 6-well plates and incubated for 24 h, then treated with *O. majorana* extract (10, 100, 200 µg/mL). Mitomycin-C (0.2 µg/mL) served as the positive control and culture medium as the negative control. After 44 h, cytochalasin B (6 µg/mL) was added to block cytokinesis and promote binucleated cell formation. After a further 28 h incubation, cells were harvested by trypsinization, subjected to hypotonic treatment (0.075 M KCl, 4 °C, 5 min), and fixed three times in cold methanol/glacial acetic acid (3:1) with 1% formaldehyde in the final fixative. Slides were prepared by dropping, air-drying, and staining with 5% Giemsa (pH 6.8) for 15 min.

Micronuclei, defined as small DNA bodies separate from and morphologically similar to the main nuclei, were scored in 3000 binucleated cells per concentration on coded slides under a light microscope (400×, Olympus BX51, Olympus Corporation, Tokyo, Japan). Three slides per dose were analyzed blindly. MN frequency was expressed per thousand (‰), and results are presented as mean ± SEM from all experiments. Cell proliferation was assessed by calculating the cytokinesis-block proliferation index (CBPI) from the distribution of mono-, bi-, tri-, and tetranucleated cells, in the first 500 cells counted on each slide for each concentration, using the formula: CBPI = (M1 + 2M2 + 3M3 + 4M4)/N, where M1–M4 represent the number of cells with 1–4 nuclei, and N is the total number of viable cells scored (excluding necrotic and apoptotic cells).

### 4.6. Experimental Animals

Twenty healthy female Wistar rats (250 ± 20 g) were obtained from the Marmara University Experimental Animal Implementation and Research Center (DEHAMER). The study protocol was approved by the Marmara University Local Ethics Committee for Animal Experiments (approval no: 18.2025mar) and was conducted in accordance with the Guide for the Care and Use of Laboratory Animals. Throughout the experimental period, animals were housed under standard laboratory conditions (temperature 22–26 °C, relative humidity 55 ± 15%, and a 12 h light/dark cycle) with ad libitum access to standard rodent chow and water. Each rat was individually marked for identification and acclimated to the laboratory environment one week before the experiments.

### 4.7. Acute Oral Toxicity Assessment

Acute toxicity tests were performed in accordance with OECD Guideline 420 (*Fixed Dose Procedure*). In the preliminary phase, two rats from each group were orally administered *O. majorana* extract at a dose of 300 mg/kg body weight (BW) via intragastric gavage. Animals were monitored for signs of toxicity or mortality over a 48 h period. In the absence of adverse effects, a second set of rats received 2000 mg/kg BW. Based on the negative findings in the preliminary phase, the main study was initiated [44].

In the main study, animals were divided into three groups (*n* = 5 per group):

Control: Vehicle control.

*O. majorana* I: *O. majorana* extract was administered intragastrically at 300 mg/kg BW.

*O. majorana* II: *O. majorana* extract was administered intragastrically at 2000 mg/kg BW.

All groups were observed daily for 14 days for mortality and clinical signs, including behavioral changes, grooming activity, vocalization, skin and eye appearance, stool consistency, and signs of distress. BW, food intake, and water consumption were recorded on days 1, 7, and 14. At the end of the experiment, animals were fasted overnight (12 h), anesthetized, and sacrificed following intracardiac blood collection [46,47,48].

#### 4.7.1. Hematological and Biochemical Analysis

Blood samples (1–3 mL) were collected into K_2_EDTA tubes for hematological assessment using an automated hematology analyzer. The parameters measured included: white blood cells (WBC), red blood cells (RBC), hemoglobin (HGB), hematocrit (HCT), platelets (PLT), neutrophils (NEU), lymphocyte (LYM), monocytes (MON), eosinophils (EOS), basophils (BAS), immature granulocytes (IMG), percentage of neutrophils (NEU%), percentage of lymphocyte (LYM%), percentage of monocytes (MON%), percentage of eosinophils (EOS%), percentage of basophils (BAS%), percentage of immature granulocytes (IMG%), mean corpuscular volume (MCV), mean corpuscular hemoglobin (MCH), mean corpuscular hemoglobin concentration (MCHC), coefficient of variation in red cell distribution width (RDV-CV), mean platelet volume (MPV), platelet distribution width (PDW), procalcitonin (PCT), platelet larger cell count (PLCC), and platelet larger cell ratio (PLCR).

For biochemical analyses, 1 mL of blood was transferred to serum-separating tubes, centrifuged (3000 rpm, 10 min), and analyzed for alanine transaminase (ALT), aspartate transaminase (AST), alkaline phosphatase (ALP), urea, blood urea nitrogen (BUN), uric acid (UA), magnesium (Mg), phosphate, Ca, Na, K, Cl, lactate dehydrogenase (LDH), creatine kinase (CK), NH_3_, lipase, amylase, total cholesterol, glucose, and triglyceride [49].

#### 4.7.2. Organ Examination and Histopathology

Liver, lung, heart, kidney, spleen, ovary, cerebrum, and eye tissues were excised, visually inspected for color and texture, and weighed. Relative organ weights (%) were calculated as [50]:

$$Relative\;organ\;weight\;\left(\%\right)=\frac{Organ\;weight}{Body\;weight}\times \;100$$

Tissue samples from lung, liver, heart, kidney, spleen, cerebrum, eye, and ovary were fixed in 10% neutral buffered formalin and processed using standard histological protocols: graded ethanol dehydration, xylene clearing, paraffin embedding, sectioning at 5 µm thickness (20x, Leica RM2125RT, Leica Microsystems, Wetzlar, Germany), and staining with haematoxylin and eosin (H&E). Microscopic evaluation was performed using established semi-quantitative scoring systems in which five to ten randomly selected fields (20× magnification) from tissue sections were evaluated by an independent, blinded histologist to ensure objectivity.

Lung: intra-alveolar haemorrhage, alveolar disruption, leukocyte infiltration, wall thickening, intra-alveolar oedema (0–3) [51,52].

Liver: hepatocellular vacuolization, Kupffer cell activation, nuclear pyknosis, sinusoidal dilatation, neutrophil infiltration, fibrosis (0–3 scale) [53].

Heart: interstitial/replacement fibrosis, nuclear atypia, fiber disarrangement, sarcoplasmic rarefaction, cytoplasmic vacuolation (0–4) [54].

Kidney: tubular injury, oedema, congestion, degeneration, dilatation, hyalinization, (0–4 scale based on % area affected) [55].

Spleen: architectural integrity, follicle structure, white/red pulp alterations (0–3) [56].

Cerebrum: cortical lamination disruption, atrophy, neuronal loss (0–3) [57].

Eye: epithelial erosion, stromal oedema, inflammatory infiltration, endothelial loss (0–3) [58].

Ovary: interstitial oedema, granulosa cell degeneration, haemorrhage, PMN infiltration, vascular dilatation (0–3) [59].

### 4.8. Statistical Analysis

Data were expressed as mean ± standard deviation (SD). Statistical significance was determined using one-way ANOVA followed by Bonferroni’s post hoc test in GraphPad Prism 9. A *p*-value < 0.05 was considered statistically significant.

## 5. Conclusions

In conclusion, this study demonstrates that *O. majorana* extract exhibits a favorable safety profile with minimal acute toxicity, stable renal and hepatic function, and immunomodulatory hematological changes, while maintaining non-cytotoxic behavior in vitro. However, dose-dependent genotoxicity observed at higher concentrations highlights the necessity of cautious application. According to the Globally Harmonized System (GHS), these findings suggest classification as Category 5 for acute oral toxicity (lowest hazard) and Category 2 for germ cell mutagenicity, reflecting both its general safety and its potential genomic risks. This dual classification underscores the importance of dose management and rigorous toxicological evaluation before considering its translational use in nutraceutical or biomedical contexts.

## Figures and Tables

**Table 1:** Values of chemical compounds of plant extract.

Components	Retention Time	µg Analyte/mg Extract ± SD
Quinic acid	6.417	1.27 ± 0.05
Chlorogenic acid	19.173	3.60 ± 0.04
Apigenin-7-O-neohesperidoside	29.503	8.44 ± 0.07
Rosmarinic acid	31.667	3.43 ± 0.22
Hesperidin	36.415	0.59 ± 0.02
8-OH salvigenin	40.523	2.89 ± 0.30

**Table 2:** The genotoxic effects of *O. majorana* extracts on fibroblast cells.

		DNAT%
Control		20.28 ± 0.32
Positive Control (H_2_O_2_) (1.7 µg/mL)		65.50 ± 0.86 ***
*O. majorana* extracts	10 µg/mL	21.07 ± 0.79 ^+++^
	100 µg/mL	25.55 ± 0.58 ***^,+++^
	200 µg/mL	27.87 ± 0.72 ***^,+++^

DNAT%: Percentage of DNA in tail. Data are shown as mean ± SD. *** *p* < 0.001 compared to control group; ^+++^ *p* < 0.001 compared to positive control group.

**Table 3:** Assessment of genotoxic effects of *O. majorana* extracts on NIH3T3 cells via MN assay.

		BN	Total MN		MN/Cell Mean ± SEM	CBPI ± SEM
			(1)	(2)		
Control		3000	6	-	0.002 ± 0.001	1.63 ± 0.01
*O. majorana* extracts	10 µg/mL	3000	15	3	0.007 ± 0.001 ^+++^	1.61 ± 0.01 ^+++^
	100 µg/mL	3000	36	5	0.015 ± 0.002 **^,+++^	1.57 ± 0.01 ***^,+++^
	200 µg/mL	3000	64	8	0.027 ± 0.003 ***^,+++^	1.52 ± 0.01 ***^,+++^
Mitomysin C		3000	168	22	0.072 ± 0.005 ***	1.35 ± 0.01 ***

BN: binucleate cells; (1) Cell with one MNi; (2) Cell with two MNi; CBPI: cytokinesis-blocked proliferation index. Data are shown as mean ± SEM. ** *p* < 0.01 and *** *p* < 0.001 compared to control group; ^+++^ *p* < 0.001 compared to positive control group.

**Table 4:** BW (g), food consumption (g) and water intake (mL) by control and rats treated with *O. majorana* extract recorded during acute toxicity study.

Groups	BW (g)			Food Consumption (g)			Water Intake (mL)		
	Day 1	Day 7	Day 14	Day 1	Day 7	Day 14	Day 1	Day 7	Day 14
Control	230.40 ± 5.32	235.64 ± 4.87	243.85 ± 7.59	23.77 ± 0.25	25.43 ± 0.86	24.92 ± 1.01	15.37 ± 0.93	14.72 ± 0.47	16.71 ± 0.83
*O. majorana* I	228.54 ± 4.93	235.53 ± 5.65	245.65 ± 5.92	25.66 ± 1.20	23.95 ± 0.45	25.47 ± 2.95	15.38 ± 2.62	13.93 ± 0.99	17.67 ± 1.51
*O. majorana* II	232.43 ± 6.24	242.78 ± 5.84	250.52 ± 8.27	24.56 ± 3.24	27.54 ± 2.46	26.72 ± 0.37	15.71 ± 3.53	16.37 ± 2.52	15.72 ± 0.83

*O. majorana* I: *O. majorana* extract was administered intragastrically at 300 mg/kg BW; *O. majorana* II: *O. majorana* extract was administered intragastrically at 2000 mg/kg BW (Body weight). Values are represented as mean + SD (*n* = 5). A *p*-value less than 0.05 (*p* < 0.05) was considered statistically significant.

**Table 5:** Haematological values of control and rats treated with *O. majorana* extract measured at the end of the acute toxicity study. The haematological data was measured by Automated Hematology Analyzer (Mindray BC-6200, Mindray Bio-Medical Electronics Co., Shenzhen, China).

Parameters	Control	*O. majorana* I	*O. majorana* II
WBC (10^3^/µL)	6.20 ± 2.05	8.40 ± 0.22	5.51 ± 0.06
NEU (10^3^/µL)	1.02 ± 0.19	4.15 ± 2.74 **	1.43 ± 0.03 ^+^
LYM (10^3^/µL)	5.03 ± 1.36	7.57 ± 2.10	3.78 ± 0.02 ^+^
MON (10^3^/µL)	0.23 ± 0.08	0.79 ± 0.29 ***	0.25 ± 0.03 ^++^
EOS (10^3^/µL)	0.30 ± 0.09	0.14 ± 0.00 **	0.06 ± 0.00 ***
BAS (10^3^/µL)	0.01 ± 0.01	0.01 ± 0.004	0.01 ± 0.00 ^++^
IMG (10^3^/µL)	0.02 ± 0.01	0.06 ± 0.03 *	0.01 ± 0.00 ^+++^
NEU%	19.22 ± 3.85	28.64 ± 7.31 *	25.80 ± 0.35
LYM%	68.63 ± 4.62	63.72 ± 7.53	68.20 ± 1.03
MON%	3.89 ± 0.72	6.16 ± 0.71 **	4.45 ± 0.60 ^+^
EOS%	6.87 ± 1.79	1.32 ± 0.39 ***	1.20 ± 0.07 ***
BAS%	0.17 ± 0.07	0.10 ± 0.07	0.05 ± 0.03 *
IMG%	0.42 ± 0.136	0.46 ± 0.08	0.05 ± 0.03 *^,++^
RBC (10^6^/µL)	7.68 ± 0.48	7.86 ± 0.32	7.77 ± 0.02
HGB (g/dL)	14.07 ± 0.36	14.00 ± 0.86	13.70 ± 0.00
HCT (%)	41.26 ± 3.59	40.18 ± 2.31	40.35 ± 0.18
MCV (fL/cell)	52.74 ± 1.96	51.10 ± 1.90	51.90 ± 0.07
MCH (pg/cell)	17.80 ± 0.46	17.80 ± 0.67	17.65 ± 0.03
MCHC (g/dL)	33.68 ± 0.74	34.82 ± 0.18 ***	34.05 ± 0.12 ^+^
RDW-CV (%)	13.04 ± 1.10	14.28 ± 1.28	16.80 ± 0.00 ***^,+++^
RDW-SD (fL)	26.82 ± 1.34	29.22 ± 2.06 *	35.25 ± 0.04 ***^,+++^
PLT (10^3^/µL)	304.8 ± 220.6	934.3 ± 183.3 ***	786.0 ± 4.95 ***
MPV (fL)	8.87 ± 0.70	7.20 ± 0.32 ***	7.25 ± 0.035 ***
PDW (fL)	15.98 ± 0.53	15.20 ± 0.12 ***	15.15 ± 0.03 ***
PCT (ng/mL)	0.25 ± 0.16	0.66 ± 0.09	0.56 ± 0.01
P-LCC (10^9^/L)	50.50 ± 17.36	86.32 ± 5.02 **	77.00 ± 2.83
P-LCR (%)	21.15 ± 6.51	9.73 ± 1.92 ***	9.80 ± 0.28 ***

WBC: white blood cells; NEU: neutrophils; LYM: lymphocyte; MON: monocytes; EOS: eosinophils; BAS: basophils; IMG: immature granulocytes; NEU%: percentage of neutrophils; LYM%: percentage of lymphocyte; MON%: percentage of monocytes; EOS%: percentage of eosinophils; BAS%: percentage of basophils; IMG%: percentage of immature granulocytes; RBC: red blood cells; HGB: haemoglobin; HCT: haematocrit; MCV: mean corpuscular volume; MCH: mean corpuscular haemoglobin; MCHC: mean corpuscular haemoglobin concentration; RDW-CV: coefficient of variation in red cell distribution width; RDW-SD: standard deviation in red cell distribution width; PLT: platelet; MPV: mean platelet volume; PDW: platelet distribution width; PCT: procalcitonin; P-LCC: platelet larger cell count; P-LCR: platelet larger cell ratio; pg: pictograms. *O. majorana* I: *O. majorana* extract was administered intragastrically at 300 mg/kg BW; *O. majorana* II: *O. majorana* extract was administered intragastrically at 2000 mg/kg BW. Values are represented as mean ± SD (*n* = 5). * *p* < 0.05, ** *p* < 0.01, *** *p* < 0.001 indicate significant changes in comparison with the normal control. ^+^ *p* < 0.05, ^++^ *p* < 0.01 and ^+++^ *p* < 0.001 compared to the positive control group.

**Table 6:** Clinical biochemistry values of control and rats treated with *O. majorana* extract measured at the end of the acute toxicity study. The clinical biochemistry data was measured by Clinical Chemistry Auto-Analyzer (Roche Cobas^®^6000 C501 Modular Analyzer, Roche Diagnostics, Mannheim, Germany).

Parameters	Control	*O. majorana* I	*O. majorana* II
Liver profile			
ALT (U/L)	122.8 ± 14.36	54.20 ± 6.18 ***	51.00 ± 0.00 ***
AST (U/L)	315.2 ± 28.49	148.8 ± 28.67 ***	112.5 ± 0.35 ***
ALP (U/L)	88.80 ± 18.58	155.2 ± 12.46 ***	130.00 ± 0.71 **
Total protein (g/L)	71.28 ± 2.56	73.04 ± 1.58	74.56 ± 0.26
Albumin (g/L)	48.88 ± 3.41	45.38 ± 7.75	49.02 ± 0.04
Renal profile			
Urea (mg/dL)	46.04 ± 1.89	47.24 ± 1.46	46.06 ± 0.33
BUN (mg/dL)	21.43 ± 0.51	21.89 ± 0.84	21.23 ± 0.34
Creatine (mg/dL)	0.40 ± 0.02	0.36 ± 0.01	0.38 ± 0.01
UA (mg/dL)	2.00 ± 0.89	1.16 ± 0.29 *	1.20 ± 0.00 **
Mg	2.59 ± 0.15	2.39 ± 0.05 **	2.32 ± 0.02 ***
Phosphate	5.22 ± 0.99	4.22 ± 0.07	2.50 ± 0.02 ***
Ca (mg/dL)	10.97 ± 1.53	11.18 ± 0.28	10.90 ± 0.01
Na	143.8 ± 4.49	143.0 ± 1.41	142.5 ± 0.35
K	6.18 ± 1.15	5.41 ± 0.31	5.26 ± 0.03
Cl	105.3 ± 2.88	120.3 ± 19.96	102.8 ± 0.21 ^+^
Cardiac profile			
LDH (U/L)	2079 ± 876.8	1383 ± 475.9	968.0 ± 0.00 **
CK (U/L)	812.4 ± 229.2	1229 ± 506.2	531.2 ± 11.67
CO_2_	12.82 ± 4.04	15.48 ± 0.86	17.06 ± 0.09 *
NH_3_	312.4 ± 134.9	221.0 ± 34.65	204.2 ± 5.41
Lipase	8.18 ± 1.03	7.76 ± 0.15	7.25 ± 0.03
Amylase	2216 ± 235.4	2077 ± 479.8	1761 ± 2.61 *
Lipid profile			
Total cholesterol (mmol/L)	73.56 ± 5.94	85.08 ± 2.18	82.12 ± 0.72
Glucose (mmol/L)	153.6 ± 26.56	139.0 ± 7.97	151.5 ± 0.35
Triglyceride (mmol/L)	296.4 ± 43.28	185.5 ± 34.84 *	144.2 ± 1.12 **

ALT: alanine transaminase; AST: aspartate aminotransferase; ALP: alkaline phosphatase; BUN: blood urea nitrogen; UA: uric acid; LDH: lactate dehydrogenase; CK: creatine kinase; CO_2_: carbon dioxide; NH_3_: ammonia. *O. majorana* I: *O. majorana* extract was administered intragastrically at 300 mg/kg BW; *O. majorana* II: *O. majorana* extract was administered intragastrically at 2000 mg/kg BW; Values are represented as mean ± SD (*n* = 5). * *p* < 0.05, ** *p* < 0.01, *** *p* < 0.001 indicate significant changes in comparison with the normal control. ^+^ *p* < 0.05 compared to positive control group.

**Table 7:** The relative organ weight of the control and rats treated with *O. majorana* extract measured by the acute toxicity study.

	Control	*O. majorana* I	*O. majorana* II
Lung	0.55 ± 0.11	0.54 ± 0.17	0.55 ± 0.09
Liver	4.01 ± 1.45	4.14 ± 2.13	4.26 ± 1.86
Heart	0.46 ± 0.01	0.48 ± 0.09	0.49 ± 0.08
Spleen	0.25 ± 0.06	0.24 ± 0.07	0.26 ± 0.10
Kidney Left	0.39 ± 0.04	0.40 ± 0.02	0.40 ± 0.08
Kidney Right	0.39 ± 0.08	0.39 ± 0.15	0.39 ± 0.06
Ovary Left	0.03 ± 0.02	0.03 ± 0.01	0.03 ± 0.01
Ovary Right	0.03 ± 0.01	0.03 ± 0.02	0.03 ± 0.01

*O. majorana* I: *O. majorana* extract was administered intragastrically at 300 mg/kg BW; *O. majorana* II: *O. majorana* extract was administered intragastrically at 2000 mg/kg BW; Values are represented as mean + SD (*n* = 5). Value less than 0.05, (*p* < 0.05) significant value.

**Table 8:** Semi-quantitative histopathological scoring was performed on haematoxylin and eosin (H&E) stained sections.

	Control	*O. majorana* I	*O. majorana* II
Lung	0.3 ± 0.5	1.0 ± 0.5	1.2 ± 0.6
Liver	0.5 ± 0.4	1.5 ± 0.5	2.0 ± 0.6
Heart	0.2 ± 0.4	1.0 ± 0.6	0.8 ± 0.4
Kidney	0.7 ± 0.6	2.0 ± 0.5	2.3 ± 0.6
Spleen	0.3 ± 0.4	1.0 ± 0.5	2.0 ± 0.6
Ovary	0.6 ± 0.5	1.5 ± 0.5	2.0 ± 0.5
Cerebrum	0.5 ± 0.5	1.8 ± 0.6	2.2 ± 0.6
Eye	0.6 ± 0.5	1.5 ± 0.5	1.6 ± 0.5

Data are presented as mean ± SD (*n* = 5).

## Data Availability

All the raw data are available from the corresponding author upon reasonable request.

## Abbreviations

The following abbreviations are used in this manuscript:

BAS%	percentage of basophils
EOS%	percentage of eosinophils
IMG%	percentage of immature granulocytes
LYM%	percentage of lymphocyte
MON%	percentage of monocytes
NEU%	percentage of neutrophils
ALP	alkaline phosphatase
ALT	alanine transaminase
ANOVA	one-way analysis of variance
AST	aspartate transaminase
BAS	basophil
BHA	butylated hydroxyanisole
BHT	butylated hydroxytoluene
BUN	blood urea nitrogen
BW	body weight
CBPI	cytokinesis-block proliferation index
CK	creatine kinase
DEHAMER	The Experimental Animal Implementation and Research Center
EOS	eosinophils
GHS	Globally Harmonized System of Classification and Labelling of Chemicals
HCT	hematocrit
HGB	hemoglobin
HPLC	high-performance liquid chromatography
HPLC-DAD	high-performance liquid chromatography with diode-array detection
IMG	immature granulocyte
LDH	lactate dehydrogenase
LYM	lymphocyte
MARE	Herbarium of the Faculty of Pharmacy, Marmara University
MCH	mean corpuscular hemoglobin
MCHC	mean corpuscular hemoglobin concentration
MCV	mean corpuscular volume
MN	micronucleus
MON	monocytes
MPV	mean platelet volume
NEU	neutrophils
OECD	Organization for Economic Co-operation and Development
PCT	procalcitonin
PDW	platelet distribution width
PLCC	platelet larger cell count
PLCR	platelet larger cell ratio
PLT	platelet
RBCs	red blood cells
RDV-CV	coefficient of variation in red cell distribution width
SD	standard deviation
UA	uric acid
WBCs	white blood cells
